# Heparin Alters Viral Serpin, Serp-1, Anti-Thrombolytic Activity to Anti-Thrombotic Activity

**DOI:** 10.2174/1874091X00802010006

**Published:** 2008-02-06

**Authors:** Xing Li, Heather Schneider, Andrew Peters, Colin Macaulay, Elaine King, Yunming Sun, Liying Liu, Erbin Dai, Jennifer A. Davids, Grant McFadden, Alexandra Lucas

**Affiliations:** 1 Viron Therapeutics Inc., London, Ontario, Canada; 2 Robarts Research Institute, Division of Cardiology Department of Medicine and Department of Microbiology and Im-munology, University of Western Ontario, London, Ontario, Canada; 3 Department of Cardiovascular Medicine and Department of Molecular Genetics and Microbiology, University of Flor-ida, Gainesville, Florida, USA

**Keywords:** Serpin, heparin, myxoma virus, thrombosis, GAGs, thrombin.

## Abstract

Serine protease inhibitors (serpins) regulate coagulation and inflammation. Heparin, a glycosaminoglycan, is an important cofactor for modulation of the inhibitory function of mammalian serpins. The secreted myxoma viral serpin, Serp-1 exerts profound anti-inflammatory activity in a wide range of animal models. Serp-1 anti-inflammatory and anti-atherogenic activity is dependent upon inhibition of the uPA / uPA receptor thrombolytic complex. We demonstrate here that heparin binds to Serp-1 and enhances Serp-1 inhibition of thrombin, a human pro-thrombotic serine protease, *in vitro*, altering inhibitory activity to a more predominant anti-thrombotic activity. Heparin also facilitates the simultaneous thrombin-mediated cleavage of Serp-1 and prevents formation of a serpin-typical SDS-resistant complex, implying mutual neutralization of Serp-1 and thrombin. In a cell-based assay, heparin facilitates Serp-1 reversal of cellular activation by stabilizing cellular membrane fluidity in thrombin-activated monocytes. In conclusion, heparin and other GAGs serve as cofactors enhancing Serp-1 regulation of local thrombotic and inflammatory pathways

## INTRODUCTION

Serp-1 is a secreted protein encoded by myxoma virus, a rabbit leporipoxvirus. This viral protein belongs to the serine protease inhibitor (serpin) superfamily (McFadden J Leuko Biol 1995) [1]. Previous investigations using a Serp-1 ‘knock-out’ virus have demonstrated that Serp-1 mediates blockade of host inflammatory responses to myxoma viral infections in European rabbits (Upton Virology 1990) [2] (Macen Virology 1993) [3]. Infusion of purified Serp-1 protein also significantly reduces host inflammatory responses in a wide range of animal models, including angioplasty injury, transplant rejection and arthritis (Lucas J Immuno 2004) [4]. This viral protein is currently under investigation in clinical trials as a new class of biopharmaceutical to treat human inflammation-based disorders such as acute coronary syndromes.

The underlying mechanism of Serp-1 anti-inflammatory activity is believed to impact multiple inflammation pathways, including the inhibition of several mammalian host serine proteases, specifically tissue- and urokinase-type plasminogen activators (tPA and uPA), and plasmin. Serp-1 also binds and inhibits factor Xa and is itself cleaved and inhibited by thrombin *in vitro* under simulated physiological conditions and concentrations (Viswanathan Thromb Haemost 2006) [5] (Lomas J Biol Chem 1993) [6] (Nash J Biol

Chem 1998) [7]. Site-directed mutagenesis within the Serp-1 reactive center loop (RCL) has demonstrated that the P1-P1’ amino acid residues (Arg-Asn) as well as the P2-P7 amino acid residues are critical for Serp-1’s anti-inflammatory functions (Dai J Biol Chem 2006) [8]. In addition, expression of the uPA-receptor (uPAR) at local injury sites is a prerequisite for Serp-1 induced attenuation of vascular inflammatory responses and subsequent atheromatous plaque development (Dai J Biol Chem 2003) [9]. These data suggest that a pathway mediated by the uPA / uPAR system can be blocked by the viral serpin in host inflammation responses. While the inhibition of the uPA / uPAR complex is noted to be important to Serp-1 anti-inflammatory activity, the role of Serp-1 interactions with factor Xa and thrombin in this same anti-inflammatory activity is not defined.

Thrombin is a pluripotent enzyme that activates many cells and modulates the coagulation process involved in the inflammatory response system (Viles-Gonzalex Am Heart J 2005) [10]. Several endogenous serpins are known to inhibit thrombin activity and regulate thrombosis. Among them, anti-thrombin-III (AT-III) is the best-known serpin anti-coagulant factor that inhibits thrombin activity and prevents blood clotting (Dementiev Nat Struc Mol Biol 2004) [11] (Li Nat Struct Mol Biol 2004) [12]. In the presence of cofactor heparin, AT-III can inhibit thrombin activity with greater association rates.

Heparin is a member of a family of sulfated polysaccharides known as glycosaminoglycans (GAGs). This group of molecules includes heparan sulfate, heparin, chondroitin sulfate**, **and dermatan sulfate (Taylor FASEB J 2006) [15]. The GAGs are produced by many different cell types and interact with proteins ranging from proteases (such as those involved in blood coagulation) and extracellular signaling molecules (such as chemokines and growth factors), to lipid- and membrane-binding proteins. GAGs participate in host coagulant and inflammatory responses by interacting with the above mentioned proteins that regulate blood clotting, cell adhesion, localization, chemotaxis and migration (Handel Annu Rev Biochem 2005) [16].

The mechanism of AT-III inhibition of thrombin is mediated predominantly through binding to heparin (GAG) molecules in order to target thrombin more easily (Gettins Chem Rev 2002) [13]. This heparin / AT-III interaction is the basis for the use of heparin infusion as anti-coagulant treatments in patients with unstable coronary disease and myocardial infarctions, cerebrovascular and peripheral arterial thrombo-embolization and during vascular surgery. A heparin-binding domain has been identified in the AT-III amino acid sequence. This domain is conserved in other human serpins, such as heparin cofactor II, protease nexin 1 and plasminogen activator inhibitor type-1 (PAI-1) (Ehrlich Biochem 1991) [14]. The serpins with this conserved domain have the ability to bind to heparin. In turn, heparin acts as a cofactor to facilitate the serpin interaction with thrombin and to regulate thrombotic and inflammatory processes.

Interactions between GAGs and the viral serpin (Serp-1) and the effects of heparin on Serp-1 inhibition of serine proteinases in the thrombotic and thrombolytic cascades have not been investigated. In this study, we examine the ability of Serp-1 to bind heparin and evaluate heparin-meditated effects on Serp-1/protease interactions. This study provides new insights into the role and effect of heparin cofactors on the viral serpin-mediated inhibitory activity and is important for further elucidation of the mechanism of action and development of this new class of anti-inflammatory reagents.

## MATERIALS AND METHODS

### Proteins and Reagents

Recombinant Serp-1 protein was expressed in the transformed-CHO cell line and purified with the traditional chromatograph method as described (Nash J Biol Chem 1998) [7]. In brief, medium containing secreted viral proteins is purified by sequential column chromatographic separations as follows: 1) Hi-trap Q (Pharmacia) washed with 20mM Tris, pH8.0 and eluted with 75mM NaH2PO4, pH7.0; 2) Copper co-operated chelating column, washed with 0.1M NH4Cl and eluted with 1M NH4Cl; 3) Superdex 75 gel filtration column, buffer exchanged to 150mM NaCl, 25mM Tris, pH8.0. Eluates are analyzed by Western blot. Serpin concentration is measured by ELISA (Lau J boil Chem 2004) [24] (Rezaie Protein Sci 1998) [25] (Schechter Methods 2004) [26] (Silverman Methods 2004) [27] (Esmon Br J Haematol 2005) [28] and single band purity was > 90% by Coomassie blue staining in SDS-PAGE (sodium dodecyl sulfate polyacrylamide gel electrophoresis) (Nash J Biol Chem 1998) [7] (Dai J Biol Chem 2006) [8] (Dai J Biol Chem 2003) [9]. The concentration of purified Serp-1 was determined by absorbance at 280 nm using a molar extinction coefficient of 32,700 M^-1^ cm^-1^ (Nash J Biol Chem 1998) [7]. Five serine proteases, human tissue-type plasminogen activator (tPA) (BD Biosciences), human urokinase-type (uPA) (Sigma), plasmin (Sigma), thrombin (Sigma), and factor Xa (Chromogenix), were used in this study. Five chromogenic substrates were used in this study. They were: (1) Pefachrom-uPA for uPA; (2) Pefachrom-tPA for tPA; (3) S-2251 for plasmin; (4) S-2222 for factor Xa, and (5) S-2238 for thrombin. The first two were purchased from Pentapharm and 3, 4, and 5 were obtained from Chromogenix. Unfractionated heparin (average 25 polysaccharide units) was purchased from Leo Pharmaceuticals. Polybrene was purchased from Sigma.

### Heparin/Sepharose Bead-Binding and Electrophorseis

Heparin/Sepharose beads were prepared from the HiTrap Heparin column (Amersham). The heparin bead resin was washed three times with 1 x PBS before the pull-down experiment. Serp-1 (50 ng) in PBS buffer (pH 7.4) was added to 15 µl of the bead resin and incubated at 37°C for one hour. After incubation, 400 µl of PBS was added to wash the heparin beads. The beads were spun down at 13,000 rpm for 1 minute. The wash step was repeated three times to remove unbound Serp-1 from the resin. If an elution step was required, the Serp-1 bound beads were incubated with 100 µl of NaCl solution at different concentrations (0 M, 1 M, or 2 M). After a 10 minute incubation at room temperature, the beads were washed three times as described above. The treated heparin beads were dissolved in the SDS-PAGE loading buffer, heated at 95 °C for 3 minutes, and analyzed in 12% gels. After electrophoresis, the separated proteins on gels were transferred to nitrocellulose membrane for immunoblot analysis. The membranes were blocked with 5% skim milk in PBS buffer overnight at 4°C. A mouse anti-Serp-1 monoclonal antibody (mAb) was incubated with the membrane for Serp-1 protein detection. Goat anti-mouse IgG antibody, labeled with horseradish peroxidase, was used for detection of the mouse mAb and signal was detected on Kodak X-ray film by using the ECL Western blotting detection kit (GE Health Care Inc.).

In order to examine the formation of a Serp-1/thrombin complex band and degradation of Serp-1 bands in the presence of heparin, Serp-1 was pre-incubated with heparin before adding this mixture into the thrombin reaction. Samples were analyzed using 12% gels and stained with a Gelcode staining kit (Pierce). Gel images were recorded by the Gel-Doc system (Bio-Rad).

### Heparin / Sepharose Column Chromatography

Heparin binding affinity analysis was performed using a fast protein liquid chromatography (FPLC) system (Pharmacia LKB Biotechnology Inc.) and a 1-ml column of pre-packed Heparin/Sepharose resin (HiTrap, Amersham). 2 ml of Serp-1 solution (1 mg/ml) was loaded on the column. After washing with two column volumes of PBS, the Serp-1 protein was eluted by NaCl solution with a linear increase of concentrations from 0 M to 1 M. The elution buffer flow rate was 1 ml/min. Protein elution was monitored by detecting absorbance at 280 nm. The binding affinity was expressed as the salt elution concentration, at which the absorbance of 280 nm reached the maximum peak. The estimated salt elution concentration value was taken as the mean of three repeated experiments.

### Chromogenic Assays

Serp-1 inhibitory activity was evaluated with five proteases (tPA, uPA, plasmin, factor Xa, and thrombin) in the presence and absence of heparin. These assays were performed in microtitre plates (Nunc). Serp-1 (25 µl) was incubated with 25 µl of enzyme solution at room temperature for at least one hour to allow Serp-1 binding and inhibition of protease activity. After the incubation, 10 μl of chromogenic substrate was added into the reaction. The 96-well plate was covered and placed in a wet box (preventing evaporation) for overnight incubation at 37 °C. After a 17-hour incubation, the plate was read at 405nm by a microplate reader (Bio-Tek Instrument). The reaction buffer (100 mM NaCl, 2 mM CaCl2, 100 mM Tris-HCl, pH 7.5, Triton X-100, 0.005%) was used for dilutions of proteases, Serp-1, and chromogenic substrate. The residual protease activity was calculated by comparing to the control sample, which contained no Serp-1. In those reactions containing heparin, Serp-1 was pre-incubated with heparin solution at indicated units before the addition of protease. The enhancing effects of heparin were elucidated by comparing heparin-containing and heparin-free groups in the reactions.

To measure inhibition rates for thrombin, Serp-1 (5 µl) was added into 30 µl of the reaction buffer containing 1 mg/ml BSA. 5 µl of thrombin was added into the reaction to interact with Serp-1 for different time durations (1 minute to 20 minutes) followed by dilution (7.5 fold) with the reaction buffer containing 4 mg/ml Polybrene. Chromogenic substrate (S-2238) was added into the reaction (final concentration: 1 mM) and the absorbance at 405 nm was measured kinetically for 30 minutes with 1 minute/interval at room temperature. The remaining activity of uninhibited thrombin was calculated from the rate of chromogenic substrate hydrolysis. The second order association rate constant (Ka) of thrombin inactivation by serpins was calculated by fitting the time dependent change of thrombin activity to the following equation,

$\mathrm{Ka}=\left(-\ln\;a\right)/t\left[I\right]$

where ‘a’ is residual protease activity, ‘t’ is time, and ‘[I]’ is the serpin concentration (Rezaie J Biol Chem 1995) [17]. In all experiments, it was ensured that less than 10% of the chromogenic substrate was utilized and all inhibition assays were performed by a time course that obtained at least 50% enzyme inhibition for calculation of inhibition rates. All assays were performed in triplicate.

### Cellular Membrane Fluidity Assay

Cellular membrane fluidity provides a measure of cellular activation responses. Experiments analyzing membrane fluidity were performed according to previously described methods (Viswanathan Thromb Haemost 2006) [5]. Briefly, the human monocytic cell line (THP-1) was cultured in RPMI medium supplemented with 10% Fetal Bovine Serum (Invitrogen Canada, Burlington, Canada), Penicillin (1 U/ml) and Streptomycin (1 mg/ml). Cells at 1 x 10^6 cell/ml were labeled with 0.8 µM of BPP (1,3-Bis (1-pyrenyl) propane) (Molecular Probes Inc., Eugene, USA) three hours prior to cell activation. Cells were activated by thrombin (1 U/ml) for one hour, washed, resuspended in growth medium and treated with Serp-1 (500 ng/ml) for one hour at 37°C In the heparin-treated group, Serp-1 was mixed with 0.1 U/ml heparin prior to being adding to cells. After the end of one hour, cells were washed to remove excess fluorescent probe and monomer, and excimer fluorescence emission intensities were measured at 390nm and 485nm respectively during excitation at 320nm using a fluorescent dual wavelength reader (Fluoroskan, Thermolab system, USA). The ratio of excimer fluorescence to monomer fluorescence (Iex/Imon) is used as a measure of membrane fluidity (Viswanathan Thromb Haemost 2006) [5]. Changes in membrane fluidity among different cell treatment groups were assessed by analysis of variance (ANOVA). P-values less than or equal to 0.05 (p < 0.05) were considered significant.

### RESULTS

## Serp-1 Binds Heparin

**Fig. (1):**
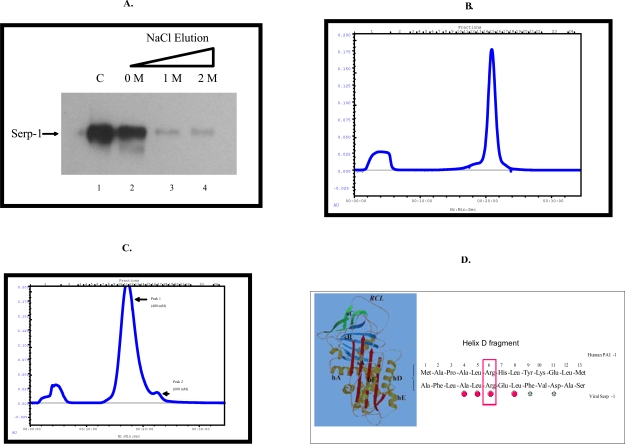
Serp-1 binds to heparin in vitro. In a pull-down experiment with heparin/sepharose beads **(A)**, Serp-1 (100 ng) was incubated with 15 µl of heparin/sepharose beads (Amersham) for one hour at 37 ^°^C. After Serp-1 bound to heparin beads, 100 µl of NaCl solution at different concentrations (0 M, 1 M, and 2 M) was added to elute Serp-1 from the heparin beads. The elution was performed at room temperature for 10 minutes. After elution, the Serp-1 heparin beads were loaded on SDS-PAGE gel for electrophoresis and followed by the immunoblot detection with the anti-Serp-1 monoclonal antibody. 100 ng Serp-1 positive control shown in lane 1. FPLC chromatograph analysis: native (B) and heat-inactivated (C) Serp-1 were loaded onto the heparin columns and eluted with a NaCl gradient. The absorbance at 280 nm was monitored. A representative elution curve is presented from three repeat experiments. X axis at bottom indicates running time of the column analysis. X axis at top indicates collecting fractions (1 ml/fraction). Y axis indicates absorbance at 280 nm. Amino acid sequence alignment (D). Human PAI-1 helix D heparin-binding domain (13 amino acid residues) aligns with the Serp-1 sequence by MacVector (version 3). The residue (Arg_6_) in Serp-1 has been mutated (boxed) to examine its significance of the heparin binding ability (see text). The identical amino acids in both sequences are dotted and the similar amino acids are starred underneath. 3-D serpin structure showing the relative positions between helix D (hD) and the reactive center loop (RCL) is adapted from Gettins (Chem Rev 2002)

Heparin-coated sepharose beads were used to examine the capacity of Serp-1 to bind individual serine proteases. Serp-1 protein binds to heparin-coated beads and is co-precipitated with beads after centrifugation (Fig. **1A**, lane 2). Furthermore, Serp-1 bound to heparin coated beads was eluted by using different concentrations of salt solution (NaCl) (Fig. **1A**, lane 3 and 4).

The affinity of Serp-1 for binding heparin was measured by using heparin-sepharose columns. Serp-1 bound to the heparin column in a neutral pH environment (pH 7.4) and was eluted as a single peak with maximum OD280 absorbency at an equivalent salt concentration of 555 mM (Fig. **1B**). In addition, the capacity of heat-inactivated Serp-1 to bind to heparin columns was assessed. To denature Serp-1 by heating, active Serp-1 solution was incubated at 75°C for 30 minutes. This denatured Serp-1 was analyzed by FPLC. The heat-treated Serp-1 was still able to bind to the heparin column, but the binding affinity was reduced from 555 mM (active Serp-1) to 400 mM NaCl (Fig. **1C**). The main peak of heat inactivated Serp-1 completely lost inhibitory activity in the uPA activity assay (Table **1**). This study indicates that the heparin binding ability of Serp-1 is independent of its inhibitory activity for uPA.

A small portion (8%) of the heat-denatured Serp-1 was also observed to be eluted at 680 mM NaCl solution (Peak 2 in Fig. **1C**). This fraction is speculated to represent polymers of Serp-1 and retained weaker inhibitory activity for uPA; 40-fold less than unheated Serp-1 (data not shown). We aligned the Serp-1 amino acid sequence with that of human PAI-1, which has a conserved heparin binding domain located in the helix D domain (Berkenpas Embo J 1995) [18]. Of thirteen amino acids, four amino acids in Serp-1 are identical to PAI-1 (31% homology) in this area (Fig. **1D**). There are three basic amino acid residues (Arg6, His7, Lys10) in the PAI-1 domain, while there is only one basic amino acid (Arg6) in the Serp-1 domain. The arginine residue is a strong basic amino acid residue and has been proved critical for heparin binding ability in both human and murine PAI-1 (Ehrlich J Biol Chem 1992) [19] (Xu J Biol Chem 2004) [20]. We performed site-directed mutagenesis at position 6 to replace this arginine with alanine. The mutated Serp-1 was still capable of binding to heparin (data not shown). These

findings suggest that the viral serpin, Serp-1, may have a different structural binding domain than PAI-1 that contributes to GAG/polyanion binding ability.

## Heparin Facilitates Interaction Between Serp-1 and Thrombin

**Fig. (2):**
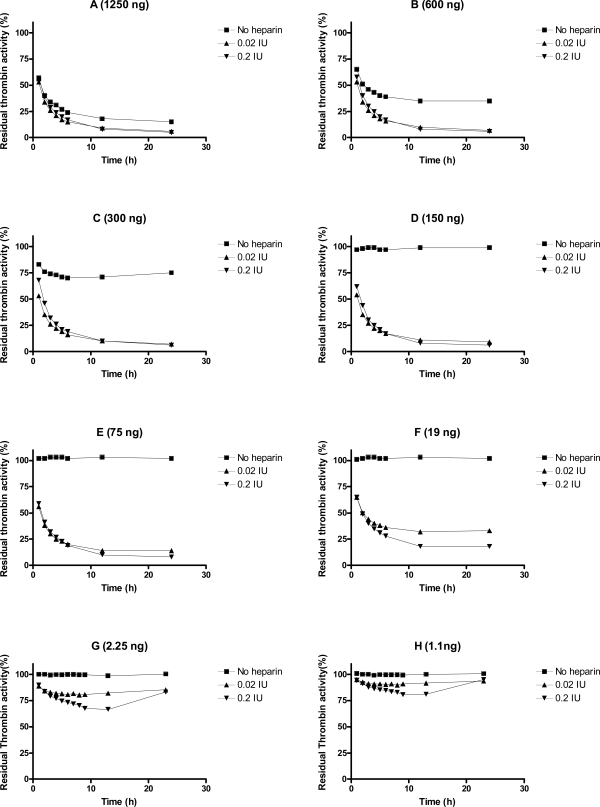
Heparin enhancing effect in the inhibition of thrombin by Serp-1. Thrombin at a fixed amount (0.002 NIH units) was incubated with Serp-1 at various amounts (A-H) in the presence or absence of heparin at 37 ^°^C for one hour. The chromogenic substrate (S-2238) was added into the reaction and color development was monitored every hour for first 6 hours and at 12 and 24 hours thereafter (X axis). The residual thrombin activity (Y axis) was expressed as percentage of the control sample, which contained no Serp-1

We evaluated heparin-mediated effects on Serp-1 inhibition of the activity of five target proteases (tPA, uPA, plasmin, factor Xa, and thrombin) by using cognate chromogenic substrate assays. No heparin enhancing effect was observed for Serp-1 inhibition of tPA, uPA, plasmin, nor factor Xa in these assays (data not shown). However, Serp-1 mediated inhibition of thrombin was enhanced by adding heparin to the Serp-1/thrombin reaction (Fig. **2**). In the absence of heparin, Serp-1 at 1250 ng, 600 ng, and 300 ng inhibited 75%, 65%, and 25% of thrombin activity, respectively (Fig. **2**. **A**, **B**, **C**, square points). Serp-1 did not inhibit thrombin activity at or below 150 ng concentrations in the reaction (Fig. **2D** to **H**, square points). With the addition of heparin, Serp-1 at low amounts could resume its inhibition of thrombin. For example, 19 ng of Serp-1 (Fig. **2F**) did not show any inhibitory activity for thrombin in the absence of heparin (square points), but was able to inhibit 75% of thrombin in the presence of heparin (0.2 IU) (triangle points), which corresponds to the ability of Serp-1 to inhibit thrombin at a concentration of 1250 ng without heparin (Compare Fig **2A** and **2F**). Thus, addition of heparin enhanced Serp-1 inhibition of thrombin approximately 66 fold in the thrombin inhibition reaction.

It is noteworthy that there was a U-shaped inhibitory curve observed in the lowest Serp-1 inhibitions (Fig. **2G** and **2H**). This indicates that the active form of thrombin is released from the Serp-1/thrombin complex after the initial inhibition. Thus, heparin may facilitate the interaction between Serp-1 and thrombin; however, heparin is unable to prevent the subsequent release of active thrombin from the Serp-1/thrombin complex.

**Fig. (3):**
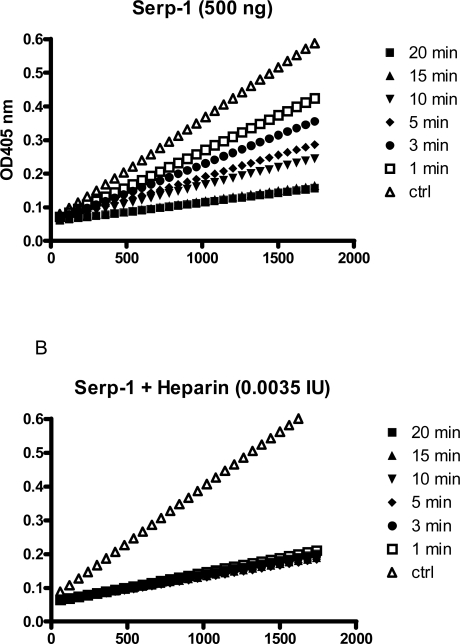
A discontinuous assay to measure thrombin inhibition rate in the absence **(A)** and presence **(B)** of heparin. A fixed amount of thrombin (0.125 NIH units) was incubated with Serp-1 (500 ng) at room temperature. At indicated time points (1, 3, 5, 10, 15, and 20 minutes), the chromogenic substrate (S-2238) containing 4 mg/ml Polybrene was added to a final concentration of 0.2 mM. Absorbance at 405 nm (Y axis) was monitored by kinetic measurement every 1 minute for 1800 seconds (X axis) at room temperature.Slopes represent the initial reaction velocity from residual thrombin and are used to calculate Ka value using the equation described in “Materials and Methods”

Heparin also dramatically enhanced the rate of Serp-1 mediated inhibition of thrombin. For example, Serp-1 at 500 ng inhibited 86% of thrombin over a 20 minute-period (Fig. **3A**). With the addition of heparin at 0.0035 IU, the inhibition rate was greatly accelerated and the inhibition process was accomplished within one minute (Fig. **3B**). In the absence of heparin, the second order association rate constant (Ka) value was measured at 9.5 ± 4.5 x 103 by the discontinuous assay. With addition of heparin, the inhibition reaction proceeded so rapidly (< 60 seconds) that no progressive inhibition curves could be obtained for the calculation of the Ka by the current assay.

## Heparin Enhancing Effect Through a Bridging Mechanism

**Fig. (4):**
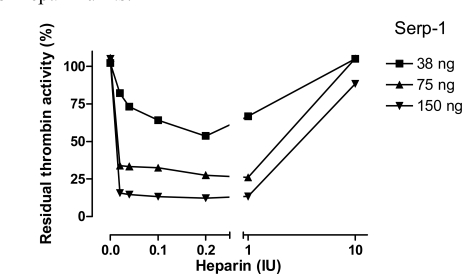
Heparin-dependent inhibition of thrombin by Serp-1. Thrombin at a fixed amount (0.002 NIH units) was incubated with Serp-1 at three non-inhibiting amounts (38, 75, and 150 ng) with increasing units of heparin (X axis). The experiment conditions were similar to those in Fig. 2 except color generation was monitored at 17 hour after the substrate was added into the reactions. The residual thrombin activity (Y axis) was expressed as percentage of the control sample, which contained no Serp-1.

To examine whether the ability of heparin to enhance Serp-1 mediated inhibition of thrombin was dependent on heparin’s presence in the reaction, the effects of five different concentrations of heparin were assessed in three non-inhibitory (38ng, 75ng, and 150ng) Serp-1 reactions (Fig. **4**) and the residual thrombin activity was plotted as a function of heparin units.

An inverted bell-shaped curve was observed in these analyses. Increasing heparin from 0.02 to 0.2 IU in the reactions produced the maximal enhancement of the Serp-1/thrombin inhibition reaction. Beyond that point, heparin did not further enhance Serp-1’s inhibitory activity. Instead, excess heparin (e.g. 10 IU) abolished Serp-1 mediated inhibition. These results strongly suggest that heparin provides a template or bridge that brings the thrombin protease and serpin into close proximity, thereby facilitating the inhibitory process. However, excess heparin is postulated to segregate thrombin and Serp-1 onto separate templates, resulting in a reduction in the capacity of Serp-1 to inhibit thrombin. This observation is consistent with a template/bridge hypothesis (Gettins Chem Rev 2002) [13].

**Fig. (5):**
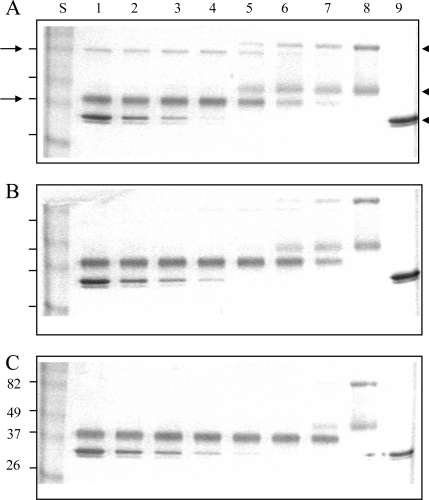
Effect of reactant concentrations of thrombin and heparin in the reaction of Serp-1 and thrombin. 10 µL samples were incubated at room temperature for 20 minutes, followed by adding the SDS-PAGE loading buffer, electrophoresis on a 12% gel, and staining with Coomassie blue kit (Gel-Code Blue, Pierce). Lanes 1 to 8 contained 1 µg Serp-1. Lanes 1 and 9 contained 2 NIH units throm-bin; lane 2, 1 NIH unit thrombin; lane 3, 0.5 NIH units thrombin; lane 4, 0.25 NIH units thrombin; lane 5, 0.12 NIH units thrombin; lane 6, 0.06 NIH units thrombin; lane 7, 0.03 NIH units thrombin. (A) There was no heparin added into the reaction. Heparin at 0.001 IU (B) and 0.01 IU (C) was added prior to the thrombin reaction. S lane contained molecular markers (kDa). Arrows at left indicate the complex band (top) and cleaved Serp-1 band (bottom). Arrowheads at right indicate the native Serp-1 and thrombin bands, respectively.

The enhancing effect of heparin in the Serp-1/thrombin interaction was also analyzed by SDS-PAGE (Fig. **5**). Native Serp-1 migrated as two bands at 96 and 47 kDa (Fig. **5** lane 8). The high molecular weight band of Serp-1 is thought to represent a dimer of the lower band. Human thrombin migrated as doublet bands at 35 kDa (Fig. **5** lane 9). When mixed for 20 minutes at room temperature, Serp-1 and thrombin formed a serpin-typical complex band at 82 kDa (arrow at left, top, Fig. **5A**). In the Serp-1/thrombin reaction, Serp-1 was also cleaved by thrombin and formed a cleaved band migrating at 37 kDa (arrow at left, bottom, Fig. **5A**). In the absence of heparin (Fig. **5A**), both the Serp-1/thrombin complex and cleaved Serp-1 bands increased along with the initial increase in thrombin in the reaction (Fig. **5A** lane 5-7). Once native Serp-1 was no longer present, there was no further increase in the levels of both cleaved Serp-1 and thrombin/Serp-1 complex (Fig. **5A** lanes 1-4). In the presence of heparin, the cleaved Serp-1 band became predominant and the complex band became invisible as heparin in the reactions increased from 0.001 IU (Fig. **5B**) to 0.01 IU (Fig. **5C**). It indicated that heparin facilitated thrombin cleavage of Serp-1 in the reaction.

**Fig. (6):**
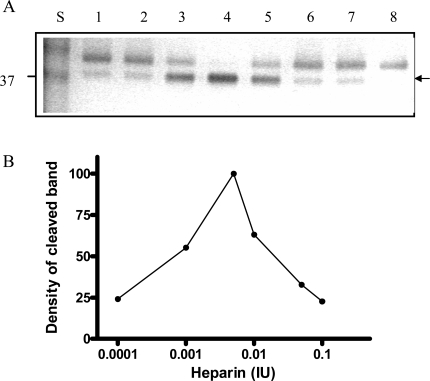
Cleavage of Serp-1 by thrombin in the presence of heparin. **(A)** The experimental conditions were identical to those in Fig. 5. Lanes 1 to 7 contained 1 µg Serp-1 and 0.03 NIH units thrombin. Lane 8 contained 1 µg Serp-1. Prior to adding thrombin into the reaction, heparin was incubated with Serp-1 at room temperature for 30 minutes. Lane 1, 0 IU heparin; lane 2, 0.0001 IU heparin; lane 3, 0.001 IU heparin; lane 4, 0.005 IU heparin; lane 5, 0.01 IU heparin; lane 6, 0.05 IU heparin; lane 7, 0.1 IU heparin. (B). Protein band density was scanned by the Gel-doc system (Bio-Rad) and plotted as a function of heparin units.

To further investigate the cleavage process of Serp-1 by thrombin in the presence of heparin, we increased the amounts of heparin in the thrombin/Serp-1 reactions and monitored the cleaved Serp-1 band by SDS-PAGE. It became obvious that formation of the cleavage band of Serp-1 was dependent on the amount of heparin units added. In this experiment, each sample contained the same amount of Serp-1 (1 µg) and thrombin (0.03 units), but different units of heparin varied from 0.0001 units (lane 2) to 0.1 units (lane 7). After incubation at room temperature for 30 minutes, the reaction was stopped and the samples were analyzed by SDS-PAGE (Fig. **6A**). The top band represents the un-cleaved, native Serp-1 monomer (47 kDa) and bottom band represents the thrombin-cleaved Serp-1 (37 kDa). The optical density of the cleaved band was measured and plotted against the heparin units presented in the reaction (Fig. **6B**). In the absence of heparin, there was only 20% of Serp-1 cleaved by thrombin in the reaction (lane 1). Increase of heparin unit from 0.0001 to 0.001 facilitated the cleavage of Serp-1 band by thrombin (lane 2 and 3). When heparin units reached at 0.005, 100% of native Serp-1 was cleaved by thrombin (lane 4). Further increase of heparin to 0.1 units did not increase the cleaved Serp-1 band. In opposite, excessive heparin prevented thrombin to cleave Serp-1 and the proportion of cleaved Serp-1 in the reaction was falling back to 20% (lane 7). Thus, the cleavage of Serp-1 by thrombin had a typical bell-shaped curve dependent on the amount of heparin presented in the reaction (Fig. **6B**).

## Cellular Membrane Fluidity Assay

**Fig. (7):**
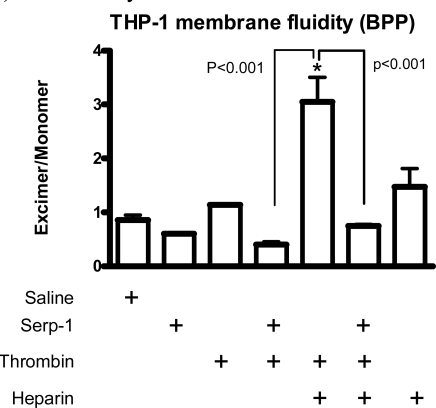
Core membrane fluidity measured using 1,3-Bis-pyrenylpropane (BPP) fluorescent probe. Membrane fluidity in the human monocytic cells (THP-1) was measured after incubation with different activators and inhibitors. Serp-1 (p=0.553), thrombin (p=0.113), or heparin (p=0.158) alone did not cause membrane fluidity changes when compared to control (saline). In the presence of heparin (0.1 U/ml), thrombin increased membrane fluidity sig-nificantly (p<0.001). Serp-1 reversed the heparin-mediated activation, bringing the fluidity level back to a normal level (p < 0.001 compared to heparin activation; p=0.793 on comparison to saline, heparin or thrombin alone without Serp-1). ^*^Significantly different (p<0.001) compared to control saline.

Changes in plasma membrane fluidity on activated cells reflect the cellular activation state (Hollan Haematologia 1996) [21]. Heparin can facilitate various circulating activators to activate immune cells. To assess the potential role of this heparin-dependent inhibition of thrombin on Serp-1 mediated anti-inflammatory activities, we measured changes in cellular core membrane fluidity after stimulation by thrombin and after treatment with Serp-1 in the absence and presence of heparin (Fig. **7**). In the THP-1 human monocyte cell line, neither thrombin (p=0.113) nor Serp-1 (p=0.553) nor heparin (p=0.158) when given alone caused significant changes in the core membrane fluidity (Fig. **7**). However, in the presence of heparin, thrombin significant increased (p<0.001) the measured membrane fluidity when compared to saline control. Serp-1, when added to thrombin treated cells, was capable of reversing thrombin mediated changes in membrane fluidity, returning the I_ex_/I_mon_ level to normal (p=0.793) (Fig. **7**). In the absence of heparin, Serp-1 was also capable of reducing the increase in membrane fluidity (I_ex_/I_mon_), but this reduction did not reach a significant level (p=0.298) in this study.

These findings indicate that Serp-1 is capable of reversing thrombin induced physiological changes in cellular activation when given in the presence of concomitant heparin treatment, as measured by altered membrane fluidity. Serp-1 thus blocked thrombin induced changes in human monocyte *in vitro* activation through a heparin dependent interaction suggesting that Serp-1 mediated anti-inflammatory activities may also depend in part on inhibition of thrombin, a prothrombotic serine protease in addition to inhibition of factor Xa and the thrombolytic proteases uPA, tPA and plasmin.

## DISCUSSION

We demonstrate here that Serp-1 binds to heparin and that the heparin-binding event facilitates the interaction between Serp-1 and a human serine protease, thrombin in the pro-thrombotic (clot forming) pathway. Thrombin is a pivotal protease that plays a central role in initiating the thrombotic process by activating various host cells and regulating coagulation, as well as inflammation. Inhibition of thrombin activity can significantly reduce thrombosis and alleviate local inflammation. This study shows that the viral serpin Serp-1, which demonstrates *in vitro* anti-thrombolysis activity, can efficiently inhibit thrombin activity in the presence of heparin. In addition, Serp-1 can also reverse monocyte activation by stabilizing cellular membrane fluidity in the presence of heparin. Thus, heparin as a cofactor can facilitate the capacity of this viral serpin to exert its potent anti-inflammatory functions through modulation of the anti-thrombotic pathway.

Heparin as a cofactor binds to several human serpins with various binding affinities, which is generally expressed as the salt concentration required for elution of a given serpin from heparin columns. High salt concentration indicates high affinity of a serpin binding to heparin. For example, antithrombin-III was eluted at 750 mM; protein C inhibitor at 350 mM; heparin cofactor at 220 mM (Pratt J Biol Chem 1992; 267(13): 8795-8801.) [22]; factor Xa at 446 mM (Pratt J Biol Chem 1992; 267(13): 8789-8794) [23], and PAI-1 at 316 mM (Ehrlich Biochem 1991) [14]. Such heparin column analysis is also used for characterization of the chemokine-GAG binding affinities (Handel Annu Rev Biochem 2005) [16]. For example, the wild type CC chemokine, MCP-1, has a heparin binding affinity measured at 486 mM (Lau J boil Chem 2004) [24]. We used heparin columns to measure Serp-1’s heparin-binding affinity. The results show that active Serp-1 has a binding affinity at 555 mM. It is not surprising that AT-III has the highest binding affinity among these serpins (750 mM). Heparin’s most prominent target is thrombin which binds glycosaminoglycan (GAG) in order to trigger its anticoagulant function in the clotting cascade. In contrast, Serp-1 has a moderate binding affinity in comparison with other human heparin-dependent serpins and GAG-binding chemokines. It is possible for Serp-1 to compete with endogenous proteins, such as clotting factors and/or chemokines, to bind to heparin and heparin-like GAG molecules *in vivo* and apply its anti-inflammatory functions through the anticoagulant pathway and GAG-mediated chemotaxis pathway.

In the anticoagulant pathway, heparin enhances the inhibitory abilities of serpins involved in the clot cascade through two mechanisms: (i) a bridging mechanism and (ii) a conformational-based mechanism (Gettins Chem Rev 2002) [13]. In the bridging mechanism, both serpin and protease simultaneously bind to a heparin molecule, allowing heparin to serve as a template to bring the two reactants together and facilitate subsequent interactions. This mechanism is characterized by a bell-shaped inhibitory curve depending on heparin concentration. We observed the typical bell-shaped curve in the heparin-assisted inhibition of thrombin by Serp-1 (Fig. **4**). Furthermore, for the first time we observed the bell-shaped curve effect in the Serp-1 cleavage process caused by thrombin in the reaction. This observation confirms the mutual neutralization of Serp-1 and thrombin in the reaction. Heparin can serve as a template to facilitate both processes.

It is unknown yet whether the heparin-binding event also causes Serp-1’s conformational changes. We observed that a low molecular weight heparin, with an average length of 15 monosaccharide units, could still enhance Serp-1 inhibition of thrombin (data not shown). The absolute length of heparin oligosaccharide units required in the Serp-1/thrombin inhibition needs to be further determined.

Three steps occur consequentially when a serpin interacting with its target protease (Gettins Chem Rev 2002) [13] (Schechter Methods 2004) [26]. These steps are (i) covalent complex formation, (ii) complex breakdown, and (iii) substrate turnover. Therefore, three parameters are used to define the effectiveness of serpin inhibition of a given target protease: stoichiometry of inhibition (SI), stability of the complex (SDS-resistant band), and the second order association rate constant (Ka). An inhibitory serpin should have its SI near 1:1, form a SDS-stable complex with target protease, and have a Ka value at approximately 104 M-1 s-1 to inhibit a target protease (Silverman Methods 2004) [27]. According to these criteria, Serp-1 was initially not considered to be an inhibitor of thrombin, because there was no SDS-stable complex band observed. Only the cleaved Serp-1 appeared in SDS-PAGE analysis (Lomas J Biol Chem 1993) [6]. It was later revealed that the Serp-1 / thrombin complex did exist, but with a very short half-life time (less than 60 minutes) (Nash J Biol Chem 1998) [7]. Also, there was a high binding stoichiometry ratio (13:1) measured in the Serp-1/thrombin interaction with a second order association rate constant (Ka) toward thrombin measured at 2.6 x 104 M-1 s-1 by the “slow binding” assay (Nash J Biol Chem 1998) [7]. It has been debated that Serp-1 does not act as an efficient inhibitor for human thrombin. Discovery of the heparin-binding ability of Serp-1 has altered this argument, because heparin not only greatly enhanced Serp-1’s capacity to inhibit thrombin (Fig. **3**), but also accelerated the cleavage of Serp-1 by thrombin in the reaction (Fig. **6**). The detailed mechanism of the Serp-1/thrombin interaction, especially in the presence of heparin, will require further definition.

Inflammation and coagulation processes are linked to form an integrated host defense system (Esmon Br J Haematol 2005) [28]. Thrombin is the final protease generated in the blood coagulation cascade. It is responsible for cleaving fibrinogen to form fibrin. In addition, thrombin also activates platelets, monocytes and macrophages to initiate the cellular responses in the thrombotic process. Previous studies show that Serp-1 can attenuate platelet and monocyte adhesion to fibronectin and collagen. The secondary messages of intracellular calcium concentration and membrane fluidity also indicate that Serp-1 can reverse the innate cellular activation stimulated by several mediators including thrombin (Viswanathan Thromb Haemost 2006) [5]. In this study, we demonstrate that Serp-1 can reverse the cellular membrane fluidity level caused by thrombin stimulation in the presence of heparin (Fig. **7**). There are two possibilities to explain this observation: (i) heparin facilitates Serp-1 to inactivate thrombin activity, therefore, thrombin can no longer interact with the target cellular receptors; or (ii) heparin binds to Serp-1 and there is no excessive free heparin to stimulate thrombin activating on cells. More experiments are needed to confirm these assumptions.

Heparin and heparin-like GAGs can bind to another group of inflammation-mediators: chemokines. In the GAG-mediated chemotaxis pathway, chemokines bind to GAGs to form a gradient in order to recruit various immune cells to inflammation sites (Handel Annu Rev Biochem 2005) [16]. Since Serp-1 has an ability to bind to heparin, it is possible that Serp-1 can compete with these molecules binding to heparin and disengage the chemokine / GAG interactions, thus, reduce cellular inflammation responses through the GAG-mediated chemotaxis pathway.

In conclusion, this study confirms that viral Serp-1 is able to bind to heparin and that heparin acts as a cofactor to facilitate the interaction of Serp-1 with one of its target proteases, thrombin. The discovery of Serp-1’s heparin-binding ability opens up new lines of investigation to elucidate the mechanisms of action whereby Serp-1 mediates a potent anti-inflammatory response against the host defense system.

## Figures and Tables

**Table 1.** Heparin affinity and uPA inhibition of native and heated Serp-1 Proteins

**Samples**	Proportion^*^(%)	Heparin Affinity^*^^*^(mM NaCl)	**uPA Inhibition**
Native Serp-1			
Unbound	5	0	No
Peak 1	95	555	Yes
Heated Serp-1			
Unbound	6	0	No
Peak 1	86	400	No
Peak 2	8	680	Yes

^*^Protein concentration was measured using the Bio-Rad Protein Assay Kit ^*^^*^Heparin affinity is given as the salt concentration required for elution ^*^^*^^*^Measured in uPA activity assay
